# Comparison of different types of therapy for overactive bladder: A systematic review and network meta-analysis

**DOI:** 10.3389/fmed.2022.1014291

**Published:** 2022-10-20

**Authors:** Peng Liu, Yan Li, Benkang Shi, Qiujie Zhang, Hu Guo

**Affiliations:** Department of Urology, Qilu Hospital, Cheeloo College of Medicine, Shandong University, Jinan, China

**Keywords:** network meta-analysis, overactive bladder, sacral neuromodulation (SNM), peripheral tibial nerve stimulation, antimuscarinics, mirabegron

## Abstract

To compare the efficacy and safety of different interventions [including antimuscarinics, mirabegron, OnabotulinumtoxinA, sacral neuromodulation (SNM) and peripheral tibial nerve stimulation (PTNS)] for treating idiopathic overactive bladder (OAB). PubMed, Embase, Cochrane Library, and other sources were searched for randomized controlled trials (RCTs) comparing interventions for overactive bladder from 1 January 2000 to 19 April 2021. A systematic review and network meta-analysis were performed by two authors independently. Fifty-five RCTs involving 32,507 patients were included in this analysis. Overall, antimuscarinics, mirabegron, OnabotulinumtoxinA, sacral neuromodulation, and peripheral tibial nerve stimulation were more efficacious than placebo, and sacral neuromodulation showed the best effect for reducing micturition frequency, urgency episodes and urgency urinary incontinence episodes. OnabotulinumtoxinA was the best intervention for achieving reductions of 100 and ≥50% in the number of urinary incontinence episodes/day, and peripheral tibial nerve stimulation was the best intervention for reducing urinary incontinence episodes. Antimuscarinics, mirabegron and peripheral tibial nerve stimulation had a similar efficacy for reducing micturition frequency, urinary incontinence episodes and urgency urinary incontinence episodes. The results revealed that all interventions examined herein were efficacious for managing adult overactive bladder syndrome compared with placebo. Furthermore, sacral neuromodulation and OnabotulinumtoxinA were the most efficient treatments for overactive bladder.

**Systematic review registration:**

[https://www.crd.york.ac.uk/prospero/display_record.php?RecordID=251966], identifier [CRD42021251966].

## Introduction

Overactive bladder (OAB) was defined in 2002 by the International Continence Society as a storage symptom characterized by “urgency, with or without urgency urinary incontinence, in the absence other obvious pathology” (1). The Epidemiology of Incontinence (EPIC) study suggested that the incidence rate of overactive bladder is high in both men and women (10.8 and 12.8%, respectively) (2, 3). Overactive bladder impairs health-related quality of life (HRQoL) and creates a significant economic burden. The cost of urinary incontinence is reported to account for approximately 2% of total health care costs in the United States each year (4). The American Urological Association (AUA) guidelines recommend behavioral therapy as the first-line treatment. The first-line behavioral therapy includes bladder training and pelvic floor muscle training (5). Seyda Toprak Celenay et al. found that pelvic floor muscle training could improve sexual dysfunction, sexual satisfaction of partners, urinary symptoms, and pelvic floor muscle strength in women with overactive bladder (6). If behavioral therapy does not work, oral antimuscarinics and mirabegron are recommended as second-line treatment. Con Kelleher et al. conducted a systematic review and revealed the efficacy and tolerability of different drug treatments for overactive bladder (7). Blayne Welk et al. found that antimuscarinics may increase the risk of mortality, and a single multicenter study showed that the mortality of β3 agonist users was 20% lower than that of antimuscarinics users (8). Mirabegron and vibegron, β3 receptor agonists, significantly improved the daily number of urgency episodes and micturition without anticholinergic adverse effects (7, 9, 10). Recently, more attention has been given to the combination treatment of antimuscarinics plus mirabegron (11, 12). Christian Gratzke et al. performed a randomized, double-blind, multicenter, phase 3 trial and proved that solifenacin succinate 5 mg plus mirabegron 50 mg was well tolerated by patients and led to greater improvements in symptoms (13). When patients are refractory to first- and second-line overactive bladder treatments, clinicians can provide third-line therapy, including OnabotulinumtoxinA, sacral neuromodulation (SNM) and peripheral tibial nerve stimulation (PTNS) (3, 14, 15). Sacral neuromodulation and peripheral tibial nerve stimulation are novel modalities and have been rapidly increasing over recent years (16, 17). Limin Liao et al. confirmed the effectiveness and safety of a novel sacral stimulation system that stimulates the sacral nerve for the treatment of overactive bladder (18). Abdullah Al-Danakh et al. suggested that peripheral tibial nerve stimulation was promising in terms of efficacy, safety, and high acceptance rate (16). This therapeutic modality was derived by balancing the potential benefits to the patient with the risks, including invasiveness of the treatment, the duration and severity of potential adverse events and the reversibility of potential adverse events. Although risks are difficult to assess, the benefits are easy to evaluate because of uniform outcome indicators. Sacral neuromodulation and peripheral tibial nerve stimulation are innovative treatments that have emerged in recent years (17, 19). There are many RCTs comparing the efficacy of those interventions for overactive bladder (13, 20), but most of them focus on drug therapy, such as antimuscarinics and Mirabegron, and very few examine sacral neuromodulation and peripheral tibial nerve stimulation, which leads to inadequate awareness among clinicians about the relative effectiveness of these two innovative treatments. Direct comparisons as well as network meta-analyses that included several of these interventions have been performed (21, 22), but no network meta-analysis has been conducted to evaluate all these treatments. Therefore, in this study, we intend to perform a systematic review and network meta-analysis to guide clinical practice by comparing the relative effectiveness of different interventions for the treatment of adults with overactive bladder.

## Materials and methods

### Study design

This network meta-analysis was conducted in accordance with the Preferred Reporting Items for Systematic Review and Meta-analysis (PRISMA) extension statement for network meta-analysis (23). This study was registered with PROSPERO (CRD42021251966).

We included RCTs reported in English comparing anticholinergics, Mirabegron, OnabotulinumtoxinA, sacral neuromodulation, peripheral tibial nerve stimulation with each other or placebo in adults with overactive bladder. The inclusion criteria and exclusion criteria are summarized in Supplementary Table 1.

### Search strategy and data sources

PubMed, Embase, Cochrane Library, and other sources were searched to find relevant articles published from 1 January 2000 to 19 April 2021. For an outcome in the same trial, only the data closest to the 12-week follow-up were extracted. The detailed search strategy is shown in Supplementary Table 2.

### Study sections and data extraction

Two investigators (PL, YL) independently extracted data using a unified data extraction form, and differences were resolved by another reviewer (HG). We recorded the outcomes as close to 12 weeks as possible. The primary outcome was mean change in the frequency of micturition/day on the basis of European Medicines Agency and Food and Drug Administration guidelines for overactive bladder trials. Secondary outcomes were the proportions of patients achieving 100 and ≥50% reductions from baseline in urinary incontinence episodes/day, mean change in urgency episodes/day, mean change in urgency urinary incontinence episodes/day, and mean change in urinary incontinence episodes/day. The main characteristics of qualified trials, such as first author, publication year, methods, number of patients, inclusion and exclusion criteria, interventions and outcomes, were extracted to conduct further analysis. The risk of bias for each RCTs was assessed using the RoB (Cochrane risk-of-bias tool for randomized trials) tool, which evaluated the following aspects: randomization process, deviations from intended interventions, missing outcome data, measurement of the outcome, and selection of the reported result. Two independent researchers (PL and YL) assessed the risk of bias of all included studies according to RoB. Disagreements were resolved by consulting a third researcher (HG). A risk of bias graph is shown in Supplementary Figure 1.

### Data analysis

Pooled odds ratios (ORs) or weighted mean differences (WMDs) with 95% confidence intervals (CIs) were analyzed. Forest plots of pairwise meta-analyses were generated by Stata 16.0 (24). We assessed network connectivity using a network diagram (Stata, version 16.0). Network meta-analysis was conducted in a Bayesian framework within the GEMTC package in the R-Statistics (25) and the J.A.G.S. program as previously described (26). Consistent and inconsistent models were considered and compared using deviance information criteria (27) (Supplementary Table 3). If the difference between them was less than 5, a fixed effects model was selected. Otherwise, a random effects model was selected. We used an unrelated mean effects model to assess inconsistency by comparing the model fit and between-study variance (heterogeneity) estimate of the pairwise comparisons against the results of the consistency model. Network consistency was assessed by comparing direct evidence and indirect evidence for each comparison using a node-splitting technique (28, 29). The assessment of heterogeneity was performed by the R package “meta,” which is represented by I^2^ ranging between 0 and 100%. The ranking probabilities were also calculated using R, and the broken line graph was generated by GraphPad Prism. Sensitivity analysis was performed to test the robustness and reliability of the results by excluding studies deemed to have a high risk of bias.

## Results

### Flow diagram and studies included

**FIGURE 1:**
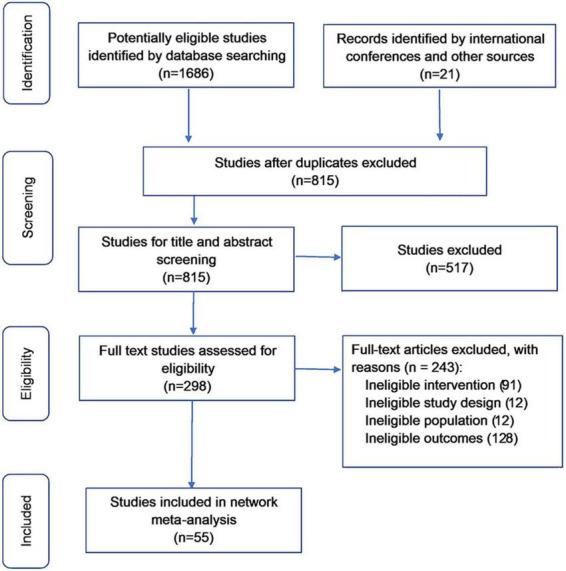
Study design.

The literature search identified 1,707 unique references, and 1,409 studies were excluded during screening (Figure 1). Of the 298 full-text articles assessed for eligibility, 243 were excluded. Overall, 55 studies (*n* = 32,507) were included in the network meta-analysis. Summaries of all included studies are shown in Supplementary Table 4.

### Pairwise meta-analyses result for different endpoints

**FIGURE 2:**
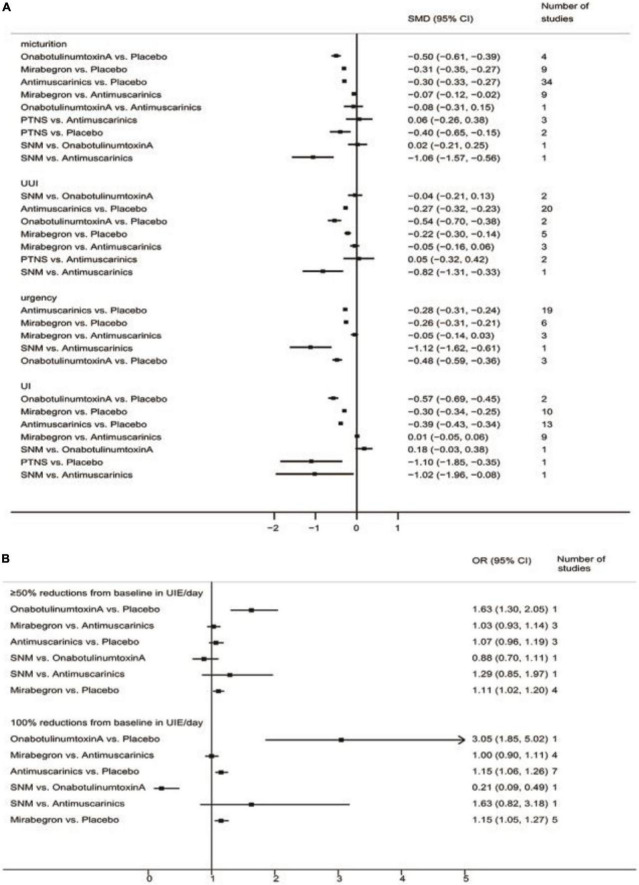
Pairwise meta-analyses result for different endpoints. **(A)** Pairwise meta-analyses result for reducing micturition frequency/day, urgency urinary incontinence episodes (UUIE)/day, urgency episodes/day, urinary incontinence episodes (UIE)/day; **(B)** Pairwise meta-analyses result for 100 and ≥50% reduction from baseline in urinary incontinence episodes (UIE)/day. SNM, sacral neuromodulation; PTNS, peripheral tibial nerve stimulation.

The pairwise meta-analyses results are shown in Figure 2.

#### Mean change in the frequency of micturition/day

The results suggested that antimuscarinics, mirabegron, OnabotulinumtoxinA, and peripheral tibial nerve stimulation were more efficacious than placebo in reducing micturition frequency. Patients receiving antimuscarinics and mirabegron showed no significant difference in reducing micturition frequency. Other direct comparisons were of limited significance due to the insufficient number of studies.

#### Mean change in urgency urinary incontinence episodes/day

The results suggested that antimuscarinics, mirabegron, and OnabotulinumtoxinA were more efficacious than placebo in reducing urgency urinary incontinence episodes. Patients receiving antimuscarinics and mirabegron showed no significant difference in reducing urgency urinary incontinence episodes. Other direct comparisons were of limited significance due to the insufficient number of studies.

#### Mean change in urgency/day

The results suggested that antimuscarinics, mirabegron, and OnabotulinumtoxinA were more efficacious than placebo in reducing urgency episodes. Patients receiving antimuscarinics and mirabegron showed no significant difference in reducing urgency episodes. Other direct comparisons were of limited significance due to the insufficient number of studies.

#### Mean change in urinary incontinence episodes/day

The results suggested that antimuscarinics, mirabegron, and OnabotulinumtoxinA were more efficacious than placebo in reducing urinary incontinence episodes. Patients receiving antimuscarinics and mirabegron showed no significant difference in reducing urinary incontinence episodes. Other direct comparisons were of limited significance due to the insufficient number of studies.

#### ≥50% reductions from baseline in urinary incontinence episodes/day

The results suggested that mirabegron was more efficacious than placebo in achieving ≥50% reductions from baseline in urinary incontinence episodes/day. There was no difference in patients receiving antimuscarinics and mirabegron, antimuscarinics and placebo. Other direct comparisons were of limited significance due to the insufficient number of studies.

#### 100% reductions from baseline in urinary incontinence episodes/day

The results suggested that antimuscarinics and mirabegron were more efficacious than placebo in achieving 100% reductions from baseline in urinary incontinence episodes/day. There was no difference in patients receiving antimuscarinics and mirabegron. Other direct comparisons were of limited significance due to the insufficient number of studies.

### Network meta-analysis of the outcomes of interests

#### Network of this study

**FIGURE 3:**
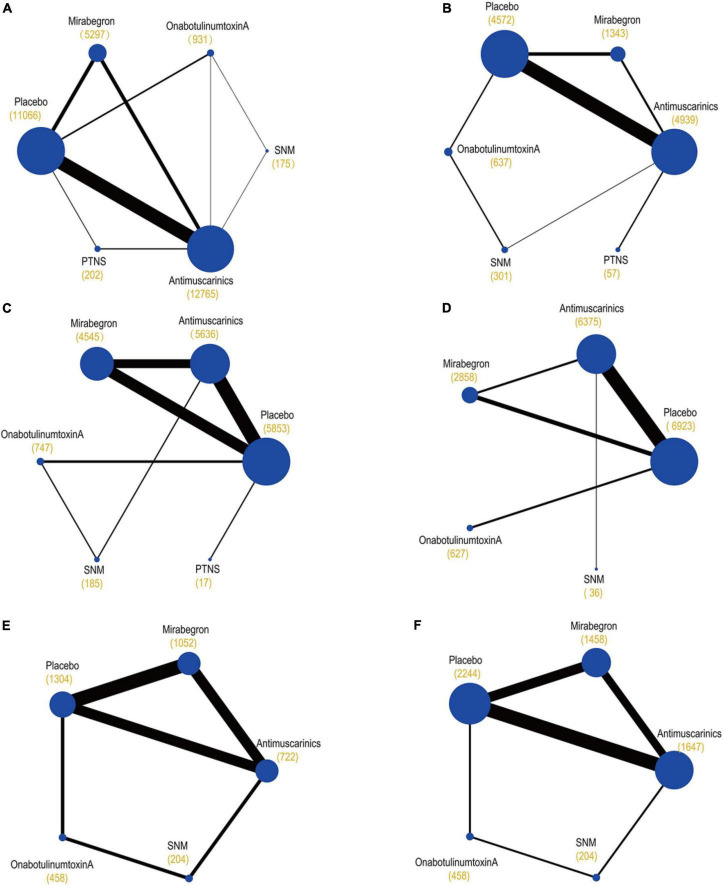
Network plots of **(A)** mean change in the frequency of micturition/day; **(B)** mean change in urgency urinary incontinence episodes (UUIE)/day; **(C)** mean change in urinary incontinence episodes (UIE)/day; **(D)** mean change in urgency/day; **(E)** ≥50% reductions from baseline in urinary incontinence episodes (UIE)/day; **(F)** 100% reductions from baseline in urinary incontinence episodes (UIE)/day. These plots were made by Stata 16.0. Each circular node represents a type of treatment. Each line shows a type of head-to-head comparison. Node size and line thickness are weighted according to the number of studies evaluating each treatment and direct comparison, respectively. The total number of participants receiving a treatment is shown in brackets. SNM, sacral neuromodulation; PTNS, peripheral tibial nerve stimulation.

The primary network including all studies (regardless of RoB) for all outcomes is presented in Figure 3.

#### Network meta-analysis

**FIGURE 4:**
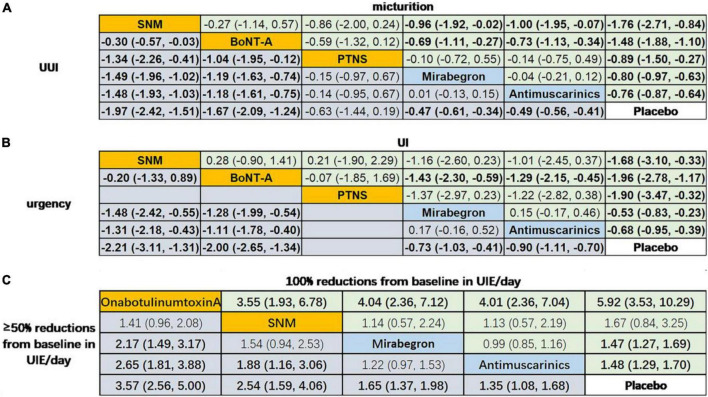
Network meta-analysis on the outcomes of interests. Data in **(A)** and **(B)** are SMD (95% CI) for the comparison of row-defining treatment versus column-defining treatment. SMD less than 0 favors upper-row treatment. Data in **(C)** are OR (95% CI) for the comparison of row-defining treatment versus column-defining treatment. OR more than 1 favors upper-row treatment. Significant results are highlighted in bold; second line treatment are highlighted in blue; third line treatment are highlighted in yellow. SNM, sacral neuromodulation; PTNS, peripheral tibial nerve stimulation.

The network meta-analysis (Figure 4) indicated that treatment with OnabotulinumtoxinA or sacral neuromodulation resulted in obviously greater mean reductions in micturition frequency, urinary incontinence episodes, urgency episodes, and urgency urinary incontinence episodes compared with all of the other interventions included in the network, and the efficacy of antimuscarinics, mirabegron and peripheral tibial nerve stimulation were similar and were better than that of placebo. In addition, the results suggested that patients receiving OnabotulinumtoxinA had the highest odds of achieving reductions of 100% and ≥50% in the number of urinary incontinence episodes/day [odds ratios relative to placebo: 5.92 (95% CI 3.53–10.29) and 3.57 (95% CI 2.56–5.00)]. Additionally, antimuscarinics or mirabegron was superior to placebo in 100 and ≥50% reductions from baseline in urinary incontinence episodes/day, respectively.

#### Rank probabilities

**FIGURE 5:**
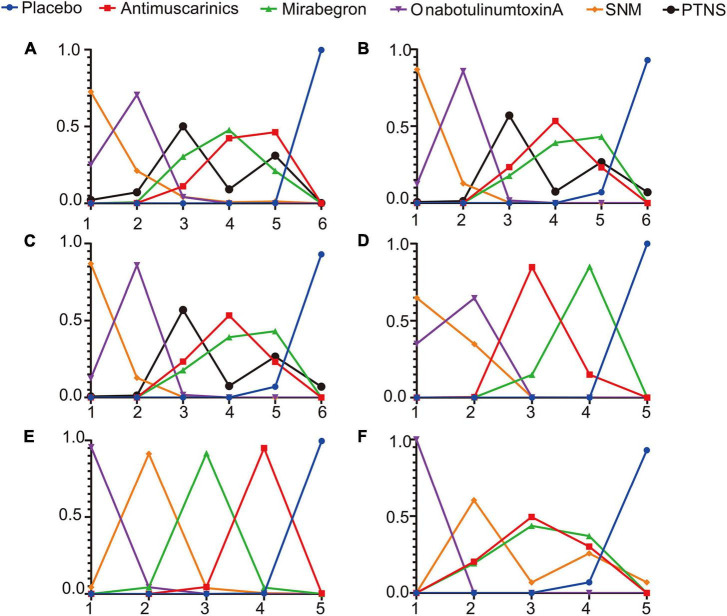
Rank probabilities of **(A)** mean change in the frequency of micturition/day; **(B)** mean change in urgency urinary incontinence episodes (UUIE)/day; **(C)** mean change in urinary incontinence episodes (UIE)/day; **(D)** mean change in urgency/day; **(E)** ≥50% reductions from baseline in urinary incontinence episodes (UIE)/day; **(F)** 100% reductions from baseline in urinary incontinence episodes (UIE)/day. The X-axis represents the ranking, and the Y-axis represents the possibility of the ranking. These plots were made by GraphPad Prism 8. SNM, sacral neuromodulation; PTNS, peripheral tibial nerve stimulation.

The Bayesian ranking probabilities of comparable treatments in different populations are shown in Figure 5.

Overall, sacral neuromodulation was most likely to be ranked first for reducing micturition frequency, urgency episodes and urgency urinary incontinence episodes, and placebo ranked lowest. The ranking results for urinary frequency reduction and urgency urinary incontinence episodes reduction were as follows: sacral neuromodulation ranked first, OnabotulinumtoxinA ranked second, peripheral tibial nerve stimulation ranked third, one of Mirabegron and Antimuscarinics ranked fourth and the other ranked fifth, and placebo ranked sixth. The ranking results for urgency episodes reduction were as follows: sacral neuromodulation ranked first, OnabotulinumtoxinA ranked second, Antimuscarinics ranked third, Mirabegron ranked fourth, and placebo ranked fifth. The ranking results for urinary incontinence episode reduction were as follows: sacral neuromodulation, OnabotulinumtoxinA and peripheral tibial nerve stimulation were the top three in no particular order, antimuscarinics ranked fourth, Mirabegron ranked fifth, and placebo ranked sixth. The ranking results for achieving reductions of 100 and ≥50% in the number of urinary incontinence episodes/day were as follows: OnabotulinumtoxinA ranked first, sacral neuromodulation ranked second, and placebo ranked lowest.

#### Consistency and inconsistency assessment

The fit of the consistency model in all comparisons was similar to or better than the fit of the inconsistency model (Supplementary Table 3). Node splitting analysis was performed to evaluate consistencies by comparing differences between the direct and indirect evidence, and the results are shown in Supplementary Table 5, which showed no significant differences in most comparisons except for placebo vs. OnabotulinumtoxinA, Anticholinergics vs. sacral neuromodulation, OnabotulinumtoxinA vs. sacral neuromodulation in the comparison of micturition, urinary incontinence episodes and 100% reductions from baseline in urinary incontinence episodes/day.

#### Sensitivity analyses

The results of the sensitivity analysis (Supplementary Figures 2 and 3) excluding 12 studies considered to have a high RoB showed little impact on the results of the network meta-analysis. The main changes in the sensitivity analysis are summarized in Supplementary Table 6.

## Discussion

In the absence of a direct head-to-head comparison of all second-line and third-line treatments recommended by AUA for adult overactive bladder symptoms, the present detailed systemic review and network meta-analysis is the first review to compare the relative effectiveness of all those interventions. Six efficacy outcomes were assessed, with each network including between 7 and 52 studies.

The results from pairwise meta-analysis revealed that all five interventions were more efficacious than placebo with regard to the outcomes included in this study. The results from the network meta-analysis indicated that OnabotulinumtoxinA and sacral neuromodulation were more efficacious than all of the other interventions in reducing micturition frequency, urinary incontinence episodes, urgency episodes and urgency urinary incontinence episodes. The efficacy of antimuscarinics, mirabegron and peripheral tibial nerve stimulation was similar. In terms of reductions of 100 and ≥50% in the number of urinary incontinence episodes/day, OnabotulinumtoxinA had the best effect.

However, this study only focuses on short-term efficacy, and the long-term results also need further discussion. Christian Gratzke et al. conducted a randomized, double-blind, multicenter, phase 3 trial to explore the long-term potential of solifenacin and mirabegron (13). They proved that patients receiving solifenacin or mirabegron showed significant improvement in reducing urinary incontinence episodes and micturition frequency. In addition, more than 40% of the patients had treatment emergent adverse events (TEAEs) after 12 months of follow-up, although the majority of the treatment emergent adverse events were mild or moderate in severity. Daisuke Kato et al. found that only 6.3% of patients experienced treatment-emergent adverse events after 1 year of mirabegron treatment. The most common treatment-emergent adverse events of antimuscarinics were dry mouth and constipation (30), which seriously affected patient compliance. Mirabegron caused an increase in blood pressure that was positively correlated with the duration of the drug (20, 31). Therefore, mirabegron is not recommended in patients with severe uncontrolled hypertension or end-stage renal disease. David Eldred-Evans et al. conducted a review and revealed that repeated OnabotulinumtoxinA injections were safe and efficacious in patients with overactive bladder (32). OnabotulinumtoxinA can cause urinary tract infections and retention of urine (22, 33), sometimes requiring catheterization to empty the bladder. Sacral neuromodulation has been shown to have long-term efficacy and subsequently exposed treatment-emergent adverse events. Bilal Kaaki et al. performed a retrospective single-institution study and proved that the success rate of sacral neuromodulation is 75% with a complication rate of 14.5% after a median follow-up of 32 months (34). Sam Tilborghs et al. conducted a systematic review of sacral neuromodulation for the treatment of overactive bladder and found that sacral neuromodulation has proven to be a safe and effective therapy in the short, medium and long term (35). Treatment-emergent adverse events of sacral neuromodulation were pain at the stimulator and lead sites, lead migration, infection, and the requirement for surgical revision (36). Sam Tilborghs et al. reported that surgical reintervention rates were high, with a median of 33.2% (range: 8–34%) in studies with at least 24 months of follow-up (35). Some studies also reported the long-term efficacy and safety of peripheral tibial nerve stimulation (37–39). Although some patients had discomfort and mild pain at the site of stimulation, as well as tingling and acid swelling of the leg, peripheral tibial nerve stimulation was safe.

In terms of cost, all third-line treatments are more expensive than drug treatments, and sacral neuromodulation is considered to be the most expensive treatment compared with other interventions in the short term (40–42), which may be an important factor affecting its application. However, sacral neuromodulation seems to be either cost saving or acceptably cost effective compared with ongoing medical therapy, peripheral tibial nerve stimulation, or OnabotulinumtoxinA in the long term (43).

There were several limitations to the current network meta-analysis study. The current study primarily compared short-term efficacy at the 12-week follow-up, and we also extracted outcome data closest to the 12-week follow-up in this study, resulting in the lack of comparison of long-term efficacy. Further research is needed to evaluate the long-term efficacy of these interventions. In addition, placebo differed in their mode of administration. In the OnabotulinumtoxinA trials, placebo was administered as a single injection, and in the mirabegron and anticholinergics trials, the placebos were administered as daily oral tablets, while in the sacral neuromodulation and peripheral tibial nerve stimulation experiment, sham stimulation was used as a placebo. To link these interventions of interest into a network, we hypothesized that different placebo treatments would be equally effective in the same population. However, one study (44) found that different administrations affected the relative efficacy of placebo for osteoarthritis of the knee, which is a limitation of this network meta-analysis.

This study further justifies the current guidelines, namely, the gradual adoption of the three-line treatments. Moreover, we should pay attention to the advantages of sacral neuromodulation in the treatment of overactive bladder, especially considering the better efficacy and fewer complications. Although behavioral therapy and drug therapy are the first- and second-line treatments for overactive bladder, the overall treatment effect is usually small. Because of limited efficacy and severe adverse effects, some patients were refractory to behavioral therapy and medical treatment. Therefore, third-line treatments should be implemented as early as possible for patients who have not benefit from medicine treatment. Besides, sacral neuromodulation should be the first choice among the third-line treatments (OnabotulinumtoxinA, sacral neuromodulation and peripheral tibial nerve stimulation) according to this study.

## Conclusion

This network meta-analysis revealed that sacral neuromodulation and OnabotulinumtoxinA achieved the best results in most outcomes at the 12-week follow-up. As there is a lack of head-to-head comparison studies among anticholinergics, mirabegron, OnabotulinumtoxinA, sacral neuromodulation, and peripheral tibial nerve stimulation for the treatment of adult overactive bladder symptoms, the present network meta-analysis provides the best available evidence for comparing these five interventions. Additionally, well-designed and head-to-head RCTs are needed to assess the efficacy and treatment-emergent adverse events of interventions in managing OAB.

## Data availability statement

The original contributions presented in this study are included in the article/Supplementary material, further inquiries can be directed to the corresponding author.

## Author contributions

PL, YL, BS, QZ, and HG: conception and design and administrative support. PL and YL: provision of study materials or patients and data analysis and interpretation. PL: collection and assembly of data. All authors: manuscript writing and final approval of manuscript.
