# Supplementary material for: Comparison of different types of therapy for overactive bladder: A systematic review and network meta-analysis
Source: Front Med (Lausanne). 2022 Oct 20;9:1014291. doi: 10.3389/fmed.2022.1014291 (PMC9633225; doi:10.3389/fmed.2022.1014291)

Supplementary materials

Supplementary Table S1. Search strategy

1. Search strategy on PubMed

(((((Cholinergic Antagonists OR (Antagonists, Cholinergic) OR Cholinergic Antagonist OR (Antagonist, Cholinergic) OR Cholinergic-Blocking Agents OR (Agents, Cholinergic-Blocking) OR Cholinergic Blocking Agents OR Cholinolytics OR Acetylcholine Antagonists OR (Antagonists, Acetylcholine) OR Cholinergic Receptor Antagonists OR (Antagonists, Cholinergic Receptor) OR (Receptor Antagonists, Cholinergic) OR Anticholinergic Agents OR (Agents, Anticholinergic) OR Anticholinergics OR Anti-Cholinergics OR Anti Cholinergics) OR ((((((((((((((((((Botulinum Toxins, Type A) OR (Clostridium botulinum A Toxin)) OR (Botulinum Toxin A)) OR (Toxin A, Botulinum)) OR (Botulinum Neurotoxin A)) OR (Neurotoxin A, Botulinum)) OR (Botulinum A Toxin)) OR (Toxin, Botulinum A)) OR (Botulinum Toxin Type A)) OR (Botulinum Neurotoxin Type A)) OR (Clostridium Botulinum Toxin Type A)) OR (Meditoxin)) OR (Botox)) OR (Neuronox)) OR (Oculinum)) OR (Vistabex)) OR (OnabotulinumtoxinA)) OR (Onabotulinumtoxin A)) OR (Vistabel))) OR ((((((Mirabegron) OR (Betmiga)) OR (2-(2-aminothiazol-4-yl)-4'-(2-((2-hydroxy-2-phenylethyl)amino)ethyl)acetanilide)) OR (Betanis)) OR (YM 178)) OR (YM-178))) OR (sacral neuromodulation)) OR (peripheral tibial nerve stimulation)) AND ((((((((Overactive bladder)) OR (Overactive Urinary Bladder)) OR (Bladder, Overactive)) OR (Overactive Detrusor)) OR (Detrusor, Overactive)) OR (Overactive Detrusor Function)) OR (Detrusor Function, Overactive)) Filters: Randomized Controlled Trial Sort by: Most Recent **Filters:** Randomized Controlled Trial **Sort by:** Most Recent

2. Search strategy on Cochrane library

ID	Search	Hits
#1	MeSH descriptor: [Cholinergic Antagonists] explode all trees	1323
#2	MeSH descriptor: [Adrenergic beta-3 Receptor Agonists] explode all trees	49
#3	(sacral neuromodulation):ti,ab,kw (Word variations have been searched)	203
#4	(peripheral tibial nerve stimulation):ti,ab,kw (Word variations have been searched)	62
#5	#1 OR #2 OR #3 OR #4	1612
#6	MeSH descriptor: [Urinary Bladder, Overactive] explode all trees	765
#7	MeSH descriptor: [Botulinum Toxins, Type A] explode all trees	1696
#8	#5 OR #7	3280
#9	#8 AND #6 in Trials	370

3. Search strategy on Embase

('overactive bladder'/exp OR 'bladder overactivity':ab,ti OR 'bladder, overactive':ab,ti OR 'detrusor overactivity':ab,ti OR 'overactive detrusor':ab,ti OR 'overactive urinary bladder':ab,ti OR 'urinary bladder, overactive':ab,ti) AND ('cholinergic receptor blocking agent'/exp OR 'acetylcholine receptor blocker':ab,ti OR 'acetylcholine receptor blocking agent':ab,ti OR 'acetylcholine receptor inhibitor':ab,ti OR 'achr inhibitor':ab,ti OR 'agent, parasympathicolytic':ab,ti OR 'anticholinergic':ab,ti OR 'anticholinergic agent':ab,ti OR 'anticholinergic drug':ab,ti OR 'anticholinergics':ab,ti OR 'atropinic agent':ab,ti OR 'atropinic drug':ab,ti OR 'central anticholinergic':ab,ti OR 'cholinergic antagonist':ab,ti OR 'cholinergic antagonists':ab,ti OR 'cholinergic blocker':ab,ti OR 'cholinergic blocking agent':ab,ti OR 'cholinergic blocking drug':ab,ti OR 'cholinergic receptor antagonist':ab,ti OR 'cholinergic receptor blocker':ab,ti OR 'cholinolytic agent':ab,ti OR 'h cholinoreactive cell':ab,ti OR 'meta cholinoreactive cell':ab,ti OR 'parasympathetic blocker':ab,ti OR 'parasympathetic blocking agent':ab,ti OR 'parasympathicolytic agent':ab,ti OR 'parasympathicolytic drug':ab,ti OR 'parasympatholytic':ab,ti OR 'parasympatholytic agent':ab,ti OR 'parasympatholytic drug':ab,ti OR 'parasympatholytics':ab,ti OR 'parasympaticolytic agent':ab,ti OR 'parasympatolytic agent':ab,ti OR 'beta 3 adrenergic receptor stimulating agent'/exp OR 'adrenergic beta 3 agonist':ab,ti OR 'adrenergic beta 3 agonists':ab,ti OR 'adrenergic beta 3 receptor agonist':ab,ti OR 'adrenergic beta 3 receptor agonists':ab,ti OR 'adrenergic beta-3 agonist':ab,ti OR 'adrenergic beta-3 agonists':ab,ti OR 'adrenergic beta-3 receptor agonist':ab,ti OR 'adrenergic beta-3 receptor agonists':ab,ti OR 'beta 3 adrenergic agonist':ab,ti OR 'beta 3 adrenergic receptor agonist':ab,ti OR 'beta 3 adrenergic receptor stimulant':ab,ti OR 'beta 3 adrenergic receptor stimulator':ab,ti OR 'beta 3 adrenergic stimulant':ab,ti OR 'beta 3 adrenergic stimulating agent':ab,ti OR 'beta 3 adrenergic stimulator':ab,ti OR 'beta 3 adrenoceptor agonist':ab,ti OR 'beta 3 adrenoceptor stimulant':ab,ti OR 'beta 3 adrenoceptor stimulating agent':ab,ti OR 'beta 3 adrenoceptor stimulator':ab,ti OR 'botulinum toxin a'/exp OR 'abobotulinum toxin a':ab,ti OR 'abobotulinumtoxin a':ab,ti OR 'abobotulinumtoxina':ab,ti OR 'agn 151607':ab,ti OR 'agn151607':ab,ti OR 'azzalure':ab,ti OR 'bocouture':ab,ti OR 'bont a':ab,ti OR 'bont a ds':ab,ti OR 'bont serotype a':ab,ti OR 'botox':ab,ti OR 'botox (100 u) injection':ab,ti OR 'botox (oculinum)':ab,ti OR 'botox 100e':ab,ti OR 'botox a':ab,ti OR 'botox cosmetic':ab,ti OR 'botulin a':ab,ti OR 'botulin toxin a':ab,ti OR 'botulinium a toxin':ab,ti OR 'botulinum a exotoxin':ab,ti OR 'botulinum a toxin':ab,ti OR 'botulinum neurotoxin a':ab,ti OR 'botulinum neurotoxin type a':ab,ti OR 'botulinum toxin type a':ab,ti OR 'botulinum toxins, type a':ab,ti OR 'btxa':ab,ti OR 'clostridium botulinum a toxin':ab,ti OR 'clostridium botulinum

endotoxin':ab,ti OR 'clostridium botulinum neurotoxin a':ab,ti OR 'clostridium botulinum neurotoxin type a':ab,ti OR 'clostridium botulinum toxin type a':ab,ti OR 'clostridium botulinum type a neurotoxin':ab,ti OR 'daxibotulinum toxin a':ab,ti OR 'daxibotulinumtoxin a':ab,ti OR 'daxibotulinumtoxina':ab,ti OR 'dwp 450':ab,ti OR 'dwp450':ab,ti OR 'dyslor':ab,ti OR 'dysport':ab,ti OR 'evabotulinum toxin a':ab,ti OR 'evabotulinumtoxin a':ab,ti OR 'evabotulinumtoxina':ab,ti OR 'evosyal':ab,ti OR 'gemibotulinum toxin a':ab,ti OR 'gemibotulinumtoxin a':ab,ti OR 'gemibotulinumtoxina':ab,ti OR 'incobotulinum toxin a':ab,ti OR 'incobotulinumtoxin a':ab,ti OR 'incobotulinumtoxina':ab,ti OR 'jeuveau':ab,ti OR 'letibotulinum toxin a':ab,ti OR 'letibotulinumtoxin a':ab,ti OR 'letibotulinumtoxina':ab,ti OR 'meditoxin':ab,ti OR 'mt 10109':ab,ti OR 'mt10109':ab,ti OR 'nabota':ab,ti OR 'nivobotulinum toxin a':ab,ti OR 'nivobotulinumtoxin a':ab,ti OR 'nivobotulinumtoxina':ab,ti OR 'nt 201':ab,ti OR 'nt201':ab,ti OR 'nuceiva':ab,ti OR 'oculinum':ab,ti OR 'onabotulinum toxin a':ab,ti OR 'onabotulinumtoxin a':ab,ti OR 'onabotulinumtoxina':ab,ti OR 'onaclostox':ab,ti OR 'pm 12759':ab,ti OR 'pm12759':ab,ti OR 'prabotulinum toxin a':ab,ti OR 'prabotulinumtoxin a':ab,ti OR 'prabotulinumtoxin a xvfs':ab,ti OR 'prabotulinumtoxina':ab,ti OR 'prabotulinumtoxina xvfs':ab,ti OR 'prabotulinumtoxina-xvfs':ab,ti OR 'prosigne':ab,ti OR 'purtox':ab,ti OR 'qm 1114':ab,ti OR 'qm1114':ab,ti OR 'relabotulinum toxin a':ab,ti OR 'relabotulinumtoxin a':ab,ti OR 'relabotulinumtoxina':ab,ti OR 'reloxin':ab,ti OR 'rtt 150':ab,ti OR 'rtt150':ab,ti OR 'vistabel':ab,ti OR 'vistabex':ab,ti OR 'xeomeen':ab,ti OR 'xeomin':ab,ti OR 'sacral nerve stimulation'/exp OR 'peripheral tibial nerve stimulation') AND 'randomized controlled trial'/de

Supplementary Table S2. Inclusion and Exclusion criteria

	**Inclusion criteria**	**Exclusion criteria**
Study design	RCTs	Observation study; non-RCTs;
Patient population	Patients (≥18 years) with idiopathic OAB	Patients with OAB and UI with a known cause (e.g., surgery, pregnancy, BPH, BOO);<br>Neurogenic OAB;<br>Bladder oversensitivity or hypersensitivity;<br>Mixed populations where results not reported separately for idiopathic OAB subgroup.
Interventions	Placebo (oral);<br>Anticholinergics (oral);<br>Mirabegron (oral);<br>OnabotulinumtoxinA (intradetrusor injection);<br>Sacral neuromodulation (SNM);<br>Peripheral tibial nerve stimulation (PTNS) ;	Other interventions;<br>Combination therapy;<br>Drugs administered by other routes;

Comparator	Any of the interventions above	Any of the interventions above
Outcomes	Mean change from baseline in the number of micturition episodes/24 h;<br>Mean change from baseline in the number of UUI episodes/24 h;<br>Mean change from baseline in the number of UI episodes/24 h;<br>Mean change from baseline in the number of urgency /24 h;<br>Number of patients with zero incontinence episodes, or<br>100% reduction in incontinence episodes;<br>Number of patients with 50% reduction in mean number of incontinence episodes;<br>TEAEs;	

Supplementary Table S3. Comparisons of the Fit of Consistency and Inconsistency Models Using Deviance Information Criteria (DIC)

	model		
	Consistency Fixed	Consistency Random	Inconsistency
micturition	**282.8011**	**215.87798**	**219.9396**
UIE	**348.63325**	**111.18062**	**111.85816**
UUIE	116.08514	113.81796	**121.94798**
urgency	**119.92625**	**98.50230**	**110.07770**
50	30.81935	32.52750	33.48112
100	57.55577	54.71854	51.50829

Supplementary Table S4. Key study features

Author, Year [ref.]	Trial Registration	Study design	Participants	Interventions	Outcomes	Duration
Abrams, P.2016	NCT01340027	randomized, double-blind	Male and female patients aged ≥18 years with OAB for ≥3 months	Solifenacin 2.5+ Mirabegron 25 mg（n=146）; Solifenacin 5+ Mirabegron 25 mg（n=141）; Solifenacin 10+ Mirabegron 25 mg（n=78）; Solifenacin 2.5+ Mirabegron 50 mg（n=147）; Solifenacin 5+ Mirabegron 50 mg（n=150）; Solifenacin 10+ Mirabegron 50 mg（n=80）; Solifenacin 2.5 mg（n=77）; Solifenacin 5 m（n=150）; Solifenacin 10 mg, （n=76）; Mirabegron 25mg（n=76）; Mirabegron 50mg（n=77）; Placebo（n=80）	OAB-q；PPBC; HRQoL;	12-week
Amundsen, C.L. 2018		randomized	Women who experienced ≥6 UUIE on a 3-d diary and failed behavioral interventions/physical therapy and two medications	Onabotulinumtoxin A 200 U (n = 192) ; Sacral neuromodulation (n = 189);	UUI; no UUI; ≥ 75% and ≥50% UUI reduction; OAB-SF; UDI-SF; PGI-I; OAB-SATq; Incontinence Impact Questionnaire; AEs；	6 months
Bray, R. 2017	NCT00137397	randomized, double-blind	Women were eligible if aged ≥ 18 years, had OAB symptoms for at least 6 months prior to entering the study, and had a BWT of at least 5 mm and post-micturition volume of less than 50 ml at screening	TER 4 mg once daily (n = 37); Placebo once daily (n = 43);	urinary frequency/day；UI；urgency episodes；MVV;	12 weeks
Chapple, C.2014		randomized, double-blind	Incontinent OAB patients	Placebo once daily (n = 862); Mirabegron 50 mg once daily (n = 878);	Urinary frequency/day; UUI; urgency；UI	12 weeks

Author, Year [ref.]	Trial Registration	Study design	Participants	Interventions	Outcomes	Duration
**Chapple, C.2013**	**NCT00910520**	**randomized, double-blind**	**Patients with idiopathic OAB with ≥3 urgency UI episodes over 3 d and ≥8 micturition per day who were inadequately managed by anticholinergics**	**OnabotulinumtoxinA 100 U (n = 277) ;<br>Placebo (n = 271);**	**PVR ;CIC ；UI；UUI; Urinary frequency/day; Urgency; nocturia; KHQ; adverse events;**	**12 weeks**
**Chapple, C.R.2014**	**NCT00689104**	**randomized**	**Men and women aged ≥18 years with OAB symptoms for ≥3 months**	**Placebo (*n*=497);<br>Mirabegron 50 mg/day (*n*=497);<br>Tolterodine ER 4 mg/day （*n*=495）;**	**UI；UUI; Urinary frequency/day; urgency; OAB-q; HRQoL; PPBC**	**12 weeks**
**Chapple, C.R.2012**	**NCT00688688**	**randomized, double-blind**	**Patients ≥18 yr of age with OAB symptoms for ≥3 months**	**Mirabegron 50 mg(*n* = 812)；<br>Mirabegron 100 mg (*n* = 820);<br>Tolterodine ER 4 mg (*n* = 812);**	**TEAEs; OAB-q; PPBC; TS-VAS；change from baseline in key OAB symptoms;**	**12 months**
**Dubeau, C. E.2014**	**NCT00928070**	**randomized, double-blind**	**Eligible men or women were 65 years old or older with self-reported UUI symptoms for 3 or more months, a mean of 2 to 15 UUI episodes, 8 or more micturition per 24 hours on baseline 3-day bladder diary, and at least some moderate bladder related problem on the PPBC who were determined to be vulnerable by a score of 3 or more on the VES-13 at screening**	**Fesoterodine 4-8mg once daily（n=283）<br>Placebo once daily（n=283）**	**UUI; Urinary frequency/day; urgency; nocturnal urgency episodes; nocturnal micturition; 100% reduction in UI; OAB-q; OAB-S:C; OAB-S: GMS; reduction in changes in absorbent products per 24 hours**	**12 weeks**
**Gratzke. C.2018**	**NCT02045862**	**randomized, double-blind**	**Men and women with symptoms of “wet” OAB (urinary frequency and urgency with incontinence) for 3 months**	**SS 5 mg plus mirabegron 50 mg (n=1218);<br>Mirabegron 50 mg (n=306);<br>SS 5 mg (n=305);**	**TEAEs; UI; Urinary； MVV；frequency/day; PVR； 100% reduction in UI; OAB-q; TS-VAS**	**12 weeks**

Author, Year [ref.]	Trial Registration	Study design	Participants	Interventions	Outcomes	Duration
Herschorn, S.2017 (1)	NCT01972841	randomized, double-blind	Patients aged ≥18 years who had had symptoms of wet OAB (urgency, urinary frequency and UI) for ≥3 months	Solifenacin 5 mg +Mirabegron 25 mg (n=853); Solifenacin 5 mg + Mirabegron 50 mg (n=848); Placebo (n=429); Mirabegron 25 mg (n=423) Mirabegron 50 mg (n=422); Solifenacin 5 mg (n=423);	UUI; Urinary frequency/day; MVV; urgency; nocturia episodes; 100% reduction in UI; TEAEs; PVR; PPBC; laboratory parameters	12 weeks
Herschorn, S.2017 (2)	NCT01767519	randomized, double-blind	Adults with symptoms of OAB (urgency, UUI, and frequency) for ≥6 months, ≥3 UUI episodes/day without >1 UUI-free day over 3 days, ≥8 micturitions/day, had an inadequate response to/were intolerant of an anticholinergic, and willing to use CIC (if needed)	OnabotulinumtoxinA 100 U (n=145); Solifenacin 5 mg (n=151); Placebo (n=60);	UI; 100% reduction in UI; AEs; Urinary frequency/day; nocturnal micturition;	12 weeks
Kaplan, S. A.2014	NCT01302054	randomized, double-blind	Men or women aged ≥ 18 years, self-reported OAB symptoms for ≥ 6 months, and at least 'some moderate problems' reported on PPBC at screening visit	Fesoterodine (n=322); Placebo (n=320);	UUI; Urinary frequency/day; ≥ 50% or 100% reductions in UUI; PPBC; UPS; OAB-q	12 weeks
Khullar, V.2012	NCT00689104	randomized, double-blind	Men and women aged ≥18 years with symptoms of OAB for ≥3 months at screening and who had experienced an average of ≥8 micturition/24 h and ≥3 urgency episodes (with or without incontinence) during the 3-day micturition diary period at baseline	Placebo (n=494) Mirabegron 50 mg once-daily (n=493); Mirabegron 100 mg once-daily (n=496); Tolterodine extended release (ER) 4 mg (n=495);	UI; Urinary frequency/day; AEs；	12 weeks

Author, Year [ref.]	Trial Registration	Study design	Participants	Interventions	Outcomes	Duration
Yuko M 2017	NCT01502956	randomized	Women with persistent UUI symptoms despite undergoing at least one supervised behavioral or physical therapy intervention and use of ≥ 2 incontinence medications (or inability to tolerate or contraindications to the medications	Onabotulinumtoxin A (n=190); SNM (n=174);	UUI；≥75% reduction in daily UUI；OABq-SF； HUI-3；LSA；PGI-I；OAB-SATq；	6 months
Kuo, H.C. 2014	NCT01043666	randomized, double-blind	Symptoms of OAB for at least 12 weeks before initiation of the run-in period; An average of 8 micturitions/d; An average of 1 episode of urgency or urgency incontinence/d, during a 3-day micturition diary period	Placebo (n=377); Mirabegron 50 mg (n=372); Tolterodine ER 4 mg (n=377);	UI; UUI; nocturia episodes; Urinary frequency/day; urgency; MVV；QoL；KHQ；TEAEs；SAF；	12 weeks
Mueller, E. R.2019		randomized, double-blind	Patients aged ≥18 years with symptoms of “wet” OAB (urinary frequency and urgency with urgency urinary incontinence or mixed incontinence with urgency as the predominant factor) for ≥3 months	Mirabegron 50 mg (*n* = 604); Combination 5 + 50 mg (*n* = 2386); Solifenacin 5 mg (*n* = 598);	TEAEs；Vital sign；ECG；PVR；OAB-q；TS-VAS；UI; MVV； Urinary frequency/day;	12 months
Nitti, V.W. 2012	NCT00662909	randomized, double-blind	Male and female patients 18 years old or older were screened for enrollment in the study if they had OAB symptoms for 3 or more months.	Placebo (*n* = 454); Mirabegron 50 mg once daily (*n* = 442); Mirabegron 100 mg once daily (*n* = 433);	UI; Urinary frequency/day; MVV；OAB-q；HRQOL；TS-VAS； PPBC；	12 weeks
Victor W 2016	NCT00910845	randomized, double-blind	Patients 18 years old or older with idiopathic OAB who experienced 3 or more urgency UI episodes in a 3-day period and an average of 8 or more micturitions per day	Placebo (*n* = 277); OnabotulinumtoxinA 100U (*n* = 280);	I-QOL; KHQ; UI; Urinary frequency/day; urgency; AE; MVV; PVR; CIC;	12 weeks

Author, Year [ref.]	Trial Registration	Study design	Participants	Interventions	Outcomes	Duration
Yokoyama, O.2011		randomized, double-blind	Men and women 20 years old or older who had a 6-month or greater history of OAB symptoms	Placebo (n = 332);<br>Solifenacin 5 mg (n = 321); Solifenacin 10 mg (n = 309);	Urinary frequency/day; MVV; KHQ；	12 weeks
Orri, M. 2014	NCT01302938	randomized, double-blind	A resident of the United States having access and ability to use a computer with Internet access; females aged ≥21 years with self-reported OAB symptoms for ≥3 months prior to screening/enrollment assessment; mean of ≥1 UUI episode per 24 hours and ≥8 micturitions per 24 hours in the 3-day micturition diary prior to the randomization/baseline assessment; and signed informed consent	Tolterodine ER 4 mg (n = 12);<br>Placebo (n = 6);	Urinary frequency/day；TEAEs；	12 weeks
Preyer, O.2015		randomized, double-blind	Female; minimum age of 18 years; complaints of OAB dry or wet consistent with the IUGA/ICS criteria; no prior treatment with PTNS or anticholinergics	Tolterodine 2 mg twice-daily (n = 18);<br>PTNS (n = 18);	Urinary frequency/day；QOL; UI	12 weeks
Siegel, S. 2015	NCT00547378	randomized, double-blind	Diagnosis of OAB as demonstrated on a 3-day voiding diary demonstrating greater than or equal to 8 voids/day and/or by having a minimum of two involuntary leaking episodes in 72 hours; Male or female and 18 years of age or older	Antimuscarinic medication (n = 77);<br>SNM (n = 70);	OAB QOL; AE; ≥ 50% or 100% reductions in UI;	6 months

Author, Year [ref.]	Trial Registration	Study design	Participants	Interventions	Outcomes	Duration
Song, M. 2015	NCT01458197	randomized, double-blind	Adult patients with OAB for at least 6 months, with an average of ≥ 8 micturitions per day and ≥ 3 incontinence episodes or a total of ≥ 6 urgency episodes per 3 days	Tarafenacin 0.2mg (n = 77);<br>Tarafenacin 0.4 mg (n = 76);<br>Placebo (n = 72);	Urinary frequency/day；UUI; urgency; nocturia episodes；QoL；KHQ；PPBC; AEs; ECG; vital signs; PVR;	12 weeks
Torimoto, K.2016	UMIN000010060	randomized Crossover	Female patients aged 50 years with OAB, who exhibited symptoms for at least 1 month and had never received treatment for the condition	Mirabegron (50 mg per day) for 8 weeks, followed by a 2-week washout period, and then imidafenacin (0.2 mg per day) for 8 weeks (n = 46);<br>Imidafenacin (0.2 mg per day) for 8 weeks, followed by a 2-week washout period, and then mirabegron (50 mg per day) for 8 weeks (n = 45);	OABSS；nocturia episodes；Urinary frequency/day；MVV； AEs;	18 weeks
Vecchioli Scaldazza, C.2013		randomized Crossover	Women with OABS	SS 5 mg once a day for 40 days and underwent PTNS for 6 weeks after 3 months from the end of therapy (washout period) (n = 20);<br>PTNS for 6 weeks; 3 months after the end of treatment (washout period), SS 5 mg once a day for 40 days (n = 20);	Urinary frequency/day；UUI; urgency; nocturia episodes；MVV; PGI-I；OABq-SF；PPIUS;	6 weeks
Wagg, A. 2015	NCT00798434	randomized, double-blind	Men and women aged 65 and older with OAB symptoms for 3 months or longer, a mean of eight or more micturitions and three or more urgency episodes per 24 hours on a 3-day bladder diary at baseline who self-reported at least some moderate problems on PPBC and had a MMSE score of 20 or greater	Placebo (n = 396);<br>Fesoterodine (n = 398);	HRQL; nocturia episodes；Urinary frequency/day；urgency; TEAEs；PPBC；UPS；	12 weeks

Author, Year [ref.]	Trial Registration	Study design	Participants	Interventions	Outcomes	Duration
Wagg, A. 2019	NCT02216214	randomized, double-blind	Community-dwelling patients aged ≥65 yr with one or more incontinence episodes, three or more urgency episodes（PPIUS grade 3 or 4), and an average of eight or more micturition episodes per day based on a 3-d micturition diary	Mirabegron (n = 445); Placebo (n = 443);	Urinary frequency/day；UUI; urgency; UI; MVV; AEs;	12 weeks
Yamaguchi, O.2015	NCT00527033	randomized, double-blind	Patients with OAB symptoms for ≥24 weeks, ≥8 micturitions/24 h on average, and ≥1 episode of urgency and/or urgency incontinence/24 h	Mirabegron 25 mg (*n* = 211); Mirabegron 50 mg (*n* = 208); Mirabegron 100 mg (*n* = 209); Placebo (*n* = 214);	Urinary frequency/day; UUI; UI; MVV; AEs; KHQ; laboratory findings; vital signs; PVR; urgency; electrocardiogram; nocturia episodes；	12 weeks
Yamaguchi, O.2014	NCT00966004	randomized, double-blind	Adult patients experiencing OAB symptoms for ≥24 weeks. Patients with ≥ 8 micturitions/24 h and ≥1 urgency episode/24 h or ≥1 urgency incontinence episode/24 h	Placebo (*n* = 381); Mirabegron 50 mg (*n* = 380); Tolterodine 4 mg (*n* = 378);	Urinary frequency/day; UI; KHQ; nocturia episodes； urgency; UUI; MVV;	12 weeks
Yamaguchi, O.2016		randomized, double-blind	Age ≥20 years, OAB symptoms for ≥24 weeks, and an average of ≥8 micturitions daily with an average of 1 or more episode of urgency and/or urge incontinence daily for 3 days	Placebo (*n* = 164); Oxybutynin 73.5 mg (*n* = 166); Oxybutynin 105 mg (*n* = 165);	Urinary frequency/day; UI; KHQ; urgency; UUI; nocturia episodes；laboratory findings; vital signs; PVR; electrocardiogram; QOL	8 weeks

Author, Year [ref.]	Trial Registration	Study design	Participants	Interventions	Outcomes	Duration
Yoshida, M.2018	JapicCTI -152936	randomized, double-blind	Male/female aged ≥20 yr old.; Symptoms of OAB for ≥6 months; Willing and able to complete the micturition diary/questionnaires correctly, including record of volume of urine voided	Vibegron 50 mg (n = 372);<br>Vibegron 100 mg (n = 372);<br>Placebo (n = 371);<br>Imidafenacin (n =117);	TEAEs; UI; MVV; UUI; urgency; nocturia episodes; Urinary frequency/day;	12 weeks
Denys, P.2012	NCT00231491	randomized, double-blind	Patients were included if they had three or more episodes of urgency with or without UUI per 3 d; eight or more voidings per 24 h; a proven detrusor overactivity (DO); and were refractory, had contraindications to, or discontinued anticholinergics because of adverse events	Placebo (n = 31)；<br>OnabotulinumtoxinA 50 U (n= 23)；<br>OnabotulinumtoxinA 100 U (n= 23)；<br>OnabotulinumtoxinA 150 U (n= 30)；	>50% improvement in urgency and UUI；Urinary frequency/day; urgency; UUI; Urodynamic measures；I-QoL；	6 months
Dmochowski,R.R. 2010		randomized, double-blind	Men and women aged ≥18 years who reported OAB symptoms for ≥3 months before screening, recorded a mean of ≥8 micturitions per 24 hours and ≥3 urgency episodes per 24 hours in a 3-day bladder diary at baseline	Fesoterodine (n = 438);<br>Placebo (n =445);	UUI；Urinary frequency/day; urgency; UPS; PPBC; AEs; OAB-q;	12 weeks
Frenkl, T. L.2010	NCT00290563	randomized, double-blind	All patients were required to have a history of urinary urgency for at least 3 months before screening, and to meet voiding diary criteria of an average of 8 or more daily micturitions and 1 or more daily urge incontinence episodes.	Serlopitant 0.25 mg (n=110);<br>Serlopitant 1mg (n=110);<br>Serlopitant 4 mg (n=114);<br>Tolterodine 4mg (n=114);<br>Placebo (n=109);	UUI；Urinary frequency/day; urgency; AEs; UI;	8 weeks

Author, Year [ref.]	Trial Registration	Study design	Participants	Interventions	Outcomes	Duration
Gotoh, M.2011		randomized, double-blind	Male and female outpatients ≥20 years old with OAB symptoms for at least 12 weeks; Patients with ≥8 micturitions/24h and ≥1 urgency incontinence episodes/24h or ≥1 urgency episodes/24h	Propiverine（n=291）; Placebo（n=274）;	OABSS; UUI；Urinary frequency/day; urgency; QOL; KHQ；electrocardiogram; AEs; laboratory findings;	12 weeks
Herschorn, S.2010		randomized, double-blind	Men or women aged ≥18 years with self-reported OAB symptoms for ≥6 months and urinary frequency (≥8 micturitions per 24 hours) and either urinary urgency (≥6 episodes) or urgency urinary incontinence (UUI; ≥3 episodes) documented in 3-day bladder diaries	Placebo(n=124); Fesoterodine 4 mg (n=234);	UUI；Urinary frequency/day; urgency; MVV; AEs; PRV;	12 weeks
Lee, K. S.2010	NCT00903045	randomized, double-blind	Men and women aged ≥18 years who had self-reported symptoms of OAB for ≥3 months	Propiverine 20 mg once daily (n = 176); Placebo once daily (n = 88)	IUSS; UPS; urgency; MVV; Urinary frequency/day; AEs; Total volume voided/24 h;	12 weeks
Peters, K.M.2010		randomized, double-blind	Adults with overactive bladder symptoms	PTNS (n = 110); Sham stimulation (n = 110);	UUI；Urinary frequency/day; urgency; OAB-q; GRA;	12 weeks
Sand, P. K.2011		randomized, double-blind	Male and female subjects experiencing OAB for ≥6 months ； urinary frequency of ≥30 toilet voids in 3 days; ≥1 'severe' urgency severity rating in 3 days; and pure urge urinary incontinence (UUI) or mixed urinary incontinence with predominant UUI, with ≥3 UUI episodes in 3 days	Placebo (n= 58)； Trospium chloride ER (n= 85)	OAB-PGA； KHQ；OAB-q； Urinary frequency/day; urgency; MVV; TEAEs；	12 weeks

ne 4 (mg)

Author, Year [ref.]	Trial Registration	Study design	Participants	Interventions	Outcomes	Duration
Enrico Finazzi-Agrò 2010		randomized, double-blind	Female patients presenting with detrusor overactivity incontinence that did not respond to antimuscarinic therapy	PTNS (n =18);<br>Placebo (n = 17);	Urinary frequency/day; UI; MVV; AEs; QoL	12 weeks
Toglia, M.R.2010	NCT00454896	randomized, double-blind	Patients (aged ≥18 years) with OAB symptoms for ≥3 months	Placebo (n=367);<br>Solifenacin (n=372);	Warning time; UI；Urinary frequency/day; nocturia episodes; IUSS; UPS; PPBC; OAB-q;	12 weeks
Herschorn, S.2008	NCT00143377	randomized, double-blind	Patients (aged ≥18 years), ≥8 micturitions/24 h on average, and ≥3 episode of urgency or urgency incontinence/24 h	Tolterodine ER (n=410);<br>Placebo (n=207);	PPBC; OAB-q; urgency; UUI; AEs; Urinary frequency/day;	12 weeks
Homma, Y.2009		randomized, double-blind	Men and women ≥20 years, who had OAB symptoms, including urinary incontinence, urinary frequency, and urgency	Imidafenacin (n =324);<br>Propiverine (n =310);<br>Placebo (n =147);	UI; AEs; electrocardiogram; AEs; Urinary frequency/day; urgency; UUI; KHQ; laboratory findings; PVR;	12 weeks
Peters, K.M.2009		randomized, double-blind	Ambulatory adults with OAB symptoms, with or without a history of previous anticholinergic drug use, with at least 8 voids per 24 hours documented by voiding diary	Tolterodine 4 mg daily (n = 50);<br>PTNS (n = 50);	Urinary frequency/day; MVV; nocturia episodes; UUI; urgency; OAB-q;	12 weeks
Staskin, D. R2006		randomized, double-blind	Outpatient men and women aged ≥18 years with a mean of ≥8 voids/24 h, and a mean of ≥1 UI episode/24 h or a mean of ≥1 urgency episode/24 h	Placebo (n=430);<br>Solifenacin 5 mg (n=159);<br>Solifenacin 10 mg (n=452);	Urinary frequency/day; urgency; UI; KHQ; TEAEs；	12 weeks

Author, Year [ref.]	Trial Registration	Study design	Participants	Interventions	Outcomes	Duration
Rogers, R.2008	NCT00143481	randomized, double-blind	Female outpatients (aged ≥18 years) with a mean of greater than or equal to eight micturitions, ≥0.6 UUI episodes, and greater than or equal to three OAB micturitions (i.e., micturitions associated with moderate or severe urgency or UUI) per 24h as recorded in 5-day bladder diaries at baseline	Placebo (n=211); Tolterodine ER (n=202);	USS； SQOL-F；UUI; AEs; Urinary frequency/day; urgency; HAD;	12 weeks
Zát'ura, F.2010		randomized, double-blind	Participants had to be outpatients aged 18–80 yr with a diagnosis of urinary incontinence with urgency and idiopathic detrusor overactivity confirmed by urodynamic study—both documented in the medical history—and showing signs of lower urinary tract dysfunction	Cizolirtine (n=54); Placebo (n=54); Oxybutynin (n=27);	Urinary frequency/day; urgency; UUI; UI; MVV;	12 weeks
Dmochowski, R. R.2008		randomized, double-blind	Subjects aged 18 years or older with OAB of 6 months or longer duration with symptoms of urinary frequency, urgency and UUI	Trospium (n=280); Placebo (n=284);	IUSS；UUI; OAB-SCS；vital signs；urgency; Urinary frequency/day; physical examination； standard laboratory tests； AEs； MVV; 100% reductions in UUI;	12 weeks

Author, Year [ref.]	Trial Registration	Study design	Participants	Interventions	Outcomes	Duration
Jacquetin, B. 2011		randomized, double-blind	Subjects aged 18 years or older with OAB of 6 months' or longer duration with symptoms of urinary frequency, urgency and UUI	Placebo (n=51); Tolterodine 1 mg (n=97); Tolterodine 2 mg (n=103);	Urinary frequency/day; UUI; MVV; AEs;	4 weeks
Nitti, V. W.2007		randomized, double-blind	Men and women 18 years or older with OAB syndrome for 6 months or greater, including urinary frequency (8 micturitions or greater per 24 hours) and urinary urgency (6 episodes or greater during the 3-day diary period) or UUI (3 episodes or greater during the 3-day diary period)	Placebo (n=274); Fesoterodine 4 mg (n=283); Fesoterodine 8 mg (n=279);	Urinary frequency/day; urgency; UUI; MVV; vital signs; electrocardiogram; AEs; laboratory findings; PVR; nocturia episodes;	12 weeks
Staskin, D.2007		randomized, double-blind	Men and women 18 years or older with symptoms of OAB for 6 months or greater	Trospium chloride 60 mg QD (n=298); Placebo (n=303);	OAB-SCS; urgency; MVV; IUSS; frequency/day; PVR; urgency; UUI; vital signs; electrocardiogram; AEs; laboratory findings;	12 weeks
Yamaguchi, O.2007		randomized, double-blind	Men and women aged ≥20 years and with symptoms of OAB reported for ≥6 months with a mean number of voids/24 h of ≥8, ≥3 episodes of urgency and/or ≥3 episodes of urgency incontinence	Solifenacin 5 mg (n=398); Solifenacin 10 mg (n=381); Propiverine 20 mg (n=400); Placebo (n=405);	QoL; Urinary frequency/day; urgency; UUI; UI; MVV; nocturia episodes; KHQ; AEs;	12 weeks

Author, Year [ref.]	Trial Registration	Study design	Participants	Interventions	Outcomes	Duration
Zhang, Y.2019		randomized, double-blind	Age greater than 16 years; Diagnosis of OAB; Refractory to standard medical therapy;	SNM (n = 37); Anticholinergic (n = 33);	Urinary frequency/day; UI; MVV; AEs; urgency; 50% reductions in UI; OABSS;	3 months
VanKerrebroeck, P.2001		randomized, double-blind	Male and female patients, 18 years of age or older, with urinary frequency (eight or more micturitions every 24 hours), urge incontinence (five or more incontinence episodes per week), and symptoms of an overactive bladder for 6 months or longer	Tolterodine ER 4 mg (n = 507); Tolterodine IR 2 mg (n = 514); Placebo (n = 508);	Urinary frequency/day; UI; MVV; AEs;	12 weeks
Zinner, N.R.2002		randomized, double-blind	Aged 18 and older, urinary frequency (≥8 micturitions per 24 hours, on average), urge incontinence (≥5 episodes per week), symptoms of overactive bladder for 6 months or more, and ability and willingness to complete micturition charts	Tolterodine ER 4 mg (n = 293); Placebo (n = 285);	Urinary frequency/day; UI; TEAEs；electrocardiogram; AEs; laboratory findings; MVV;	12 weeks

OAB-q : Overactive Bladder Questionnaire; PPBC :Patient Perception of Bladder Condition score; HRQoL: health-related QoL; SNM: sacral neuromodulation; BTX: Onabotulinumtoxin A; OAB-SF: Overactive Bladder Questionnaire Shor Form; UDI-SF: Urinary Distress Inventory short form; OAB-SATq: Overactive Bladder Satisfaction of Treatment Questionnaire; : PGI-I：Patient Global Impression of Improvement; PVR: postvoid residual urine volume; CIC: initiation of clean intermittent catheterization; I-QOL: Incontinence Quality of Life; KHQ :King's Health Questionnaire; TS-VAS: Treatment Satisfaction Visual Analog Scale; VES-13 :Vulnerable Elders Survey; OAB-S:C :OAB Satisfaction Questionnaire: Satisfaction with OAB Control Module; OAB-S:GMS :OAB Satisfaction Questionnaire: Global Medication Satisfaction question; TEAEs :treatment emergent adverse events；PVR：postvoid residual volume； MVV：mean volume voided；UPS: Urgency Perception Scale ；HUI-3：Health Utility Index Mark-3 HUI-3；LSA ：Life Space Assessment; KHQ: King's Health Questionnaire；SAF ：Selected Cardiovascular TEAEs ；ECG ：electrocardiogram；SS：solifenacin succinate；PPIUS :Patient Perception of Intensity of Urgency

Scale； MMSE ：Mini-Mental State Examination；UPS：Urgency Perception Scale；TBS ：Treatment Benefit Scale; IUSS: Indevus Urgency Severity Scale; GRA: global response assessment; SQOL-F：Sexual Quality of Life Questionnaire； PISQ ：Pelvic Organ Prolapse/Urinary Incontinence Sexual Questionnaire ；HAD: Hospital Anxiety and Depression Scale

Supplementary Table S5. Node-splitting Analysis of Inconsistency

Nodes	Direct effect	Indirect effect	Overall	P
**mean change in the frequency of micturition/day**				
Placebo vs Anticholinergics	-0.75 (-0.87, -0.63)	-0.81 (-1.20, -0.35)	-0.76 (-0.87, -0.64)	0.793825
Placebo vs Mirabegron	-0.69 (-0.86, -0.51)	-0.85 (-1.10, -0.57)	-0.80 (-0.97, -0.63)	0.334425
Placebo vs OnabotulinumtoxinA	-1.50 (-1.90, -1.10)	-11.00 (-15.00, -6.40)	-1.50 (-1.90, -1.10)	**0.000025**
Placebo vs PTNS	-1.00 (-1.80, -0.22)	-0.71 (-1.70, 0.28)	-0.90 (-1.50, -0.27)	0.630550
Anticholinergics vs Mirabegron	-0.12 (-0.31, 0.04)	-0.03 (-0.33, 0.28)	-0.04 (-0.21, 0.12)	0.599475
Anticholinergics vs OnabotulinumtoxinA	-0.20 (-0.95, 0.56)	-0.95 (-1.40, -0.47)	-0.73 (-1.10, -0.33)	0.096150
Anticholinergics vs SNM	-10.00 (-15.00, -5.50)	-0.58 (-1.50, 0.35)	-0.99 (-1.90, -0.05)	**0.000125**
Anticholinergics vs PTNS	0.05 (-0.93, 1.00)	-0.26 (-1.10, 0.54)	-0.14 (-0.77, 0.49)	0.629325
OnabotulinumtoxinA vs SNM	0.06 (-0.78, 0.92)	-9.60 (-14.00, -5.20)	-0.26 (-1.10, 0.58)	**0.000325**
**mean change in urgency UIE/day**				
Placebo vs Anticholinergics	-0.52 (-0.64, -0.40)	-0.34 (-1.50, 0.89)	-0.51 (-0.62, -0.40)	0.761675
Placebo vs OnabotulinumtoxinA	-1.70 (-2.30, -1.00)	-1.80 (-2.80, -0.91)	-1.80 (-2.30, -1.20)	0.798175
Anticholinergics vs Mirabegron	-0.15 (-0.41, 0.09)	0.14 (-0.45, 0.75)	-0.01 (-0.22, 0.19)	0.358975
Anticholinergics vs SNM	-1.30 (-2.10, -0.41)	-1.10 (-1.90, -0.28)	-1.20 (-1.70, -0.60)	0.778625
OnabotulinumtoxinA vs SNM	0.08 (-0.31, 0.49)	-0.06 (-1.20, 1.00)	0.06 (-0.30, 0.44)	0.787075
**mean change in UIE/day**				
Placebo vs Anticholinergics	-0.69 (-1.10, -0.33)	-0.53 (-1.50, 0.45)	-0.68 (-0.95, -0.40)	0.740800
Placebo vs Mirabegron	-0.41 (-0.71, -0.10)	-0.87 (-1.50, -0.20)	-0.53 (-0.83, -0.23)	0.198100
Placebo vs OnabotulinumtoxinA	-1.80 (-2.70, -1.00)	-7.90 (-14.00, -2.30)	-2.00 (-2.80, -1.20)	**0.040575**
Anticholinergics vs Mirabegron	-0.01 (-0.36, 0.32)	0.63 (-0.02, 1.30)	0.15 (-0.16, 0.46)	0.078100
Anticholinergics vs SNM	-6.90 (-13.00, -1.10)	-0.63 (-2.10, 0.81)	-1.00 (-2.50, 0.40)	**0.037850**
OnabotulinumtoxinA vs SNM	0.52 (-0.65, 1.70)	-5.70 (-12.00, 0.22)	0.28 (-0.91, 1.40)	**0.043900**
**mean change in urgency episodes /day**				
Anticholinergics vs Mirabegron	-0.17 (-0.56, 0.23)	0.32 (-0.07, 0.77)	0.17 (-0.16, 0.52)	0.083675
**100% reductions from baseline in UIE/day**				
Placebo vs Anticholinergics	0.38 (-0.04, 0.76)	-1.40 (-3.50, 0.70)	0.38 (0.08, 0.64)	0.087700
Placebo vs OnabotulinumtoxinA	1.50 (0.76, 2.20)	3.30 (1.80, 4.80)	1.80 (1.10, 2.60)	**0.030600**
Anticholinergics vs Mirabegron	-0.02 (-0.51, 0.40)	0.01 (-0.99, 1.00)	-0.03 (-0.39, 0.27)	0.917625
Anticholinergics vs SNM	0.89 (-0.11, 1.90)	-0.86 (-2.20, 0.37)	0.19 (-0.63, 1.10)	**0.034300**
OnabotulinumtoxinA vs SNM	-1.90 (-3.00, -0.97)	-0.15 (-1.40, 1.10)	-1.30 (-2.10, -0.49)	**0.028775**
**≥50% reductions from baseline in UIE/day**				
Placebo vs Anticholinergics	0.11 (-1.30, 1.00)	-0.26 (-3.60, 3.00)	0.27 (-0.25, 0.58)	0.762950
Placebo vs OnabotulinumtoxinA	1.20 (0.50, 1.90)	1.70 (0.33, 3.00)	1.30 (0.74, 1.90)	0.416425
Anticholinergics vs Mirabegron	0.13 (-0.66, 0.81)	0.83 (-1.60, 3.30)	0.20 (-0.21, 0.58)	0.418400
Anticholinergics vs SNM	1.00 (-0.04, 2.10)	0.48 (-0.54, 1.70)	0.69 (0.06, 1.50)	0.421100
OnabotulinumtoxinA vs SNM	-0.44 (-1.10, 0.26)	0.09 (-1.20, 1.40)	-0.32 (-0.94, 0.27)	0.401575

Supplementary Table S6. Sensitivity Analysis Description

Sensitivity analysis excluding 12 studies considered to have a high RoB		
**Outcome**	**Changes in Ranking probabilities**	**Variations of SMD/CrI or OR/CrI from original analysis to sensitivity analysis**
**Micturition frequency**	1. SNM 2. OnabotulinumtoxinA 3. PTNS 4. Mirabegron 5. Antimuscarinics 6. Placebo (No change with the original result)	OnabotulinumtoxinA vs PTNS：-0.59（-1.32，0.12）to -0.83（-1.65，-0.03）
**UUIE**	1. OnabotulinumtoxinA (↑) 2. SNM (↓) 3. PTNS 4. Antimuscarinics (↑) 5. Mirabegron (↓) 6. Placebo	SNM vs OnabotulinumtoxinA：-0.30（-0.57，-0.03）to 0.43（-0.10, 0.97）；SNM vs PTNS: -1.34（-2.26，-0.41）to -0.88（-1.89，0.09）
**UIE**	1. PTNS 2. OnabotulinumtoxinA 3. SNM 4. Antimuscarinics 5. Mirabegron 6. Placebo (No change with the original result)	No change with the original result
**Urgency**	1. SNM 2. Mirabegron (↑) 3. OnabotulinumtoxinA (↓) 4. Antimuscarinics (↓) 5. Placebo	No change with the original result
**≥50% reductions from baseline in UIE/day**	1. OnabotulinumtoxinA 2. SNM 3. Mirabegron 4. Antimuscarinics 5. Placebo (No change with the original result)	No change with the original result
**100% reductions from baseline in UIE/day**	1. OnabotulinumtoxinA 2. Antimuscarinics (↑) 3. Mirabegron (↑) 4. Placebo (↑) 5. SNM (↓)	SNM vs Antimuscarinics: 1.88（1.16,3.06） to 1.53（0.83,2.79）

Supplementary Figure S1. Summary of Results from Bias Risk Assessment of Studies

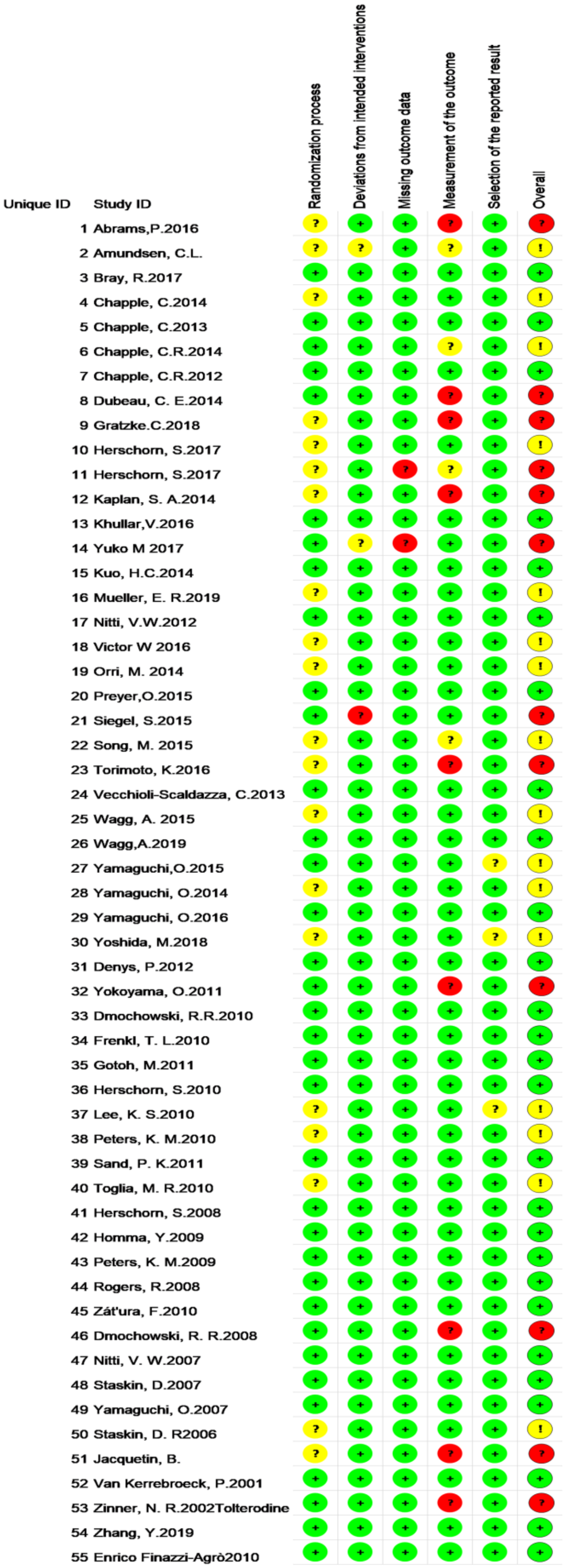

Supplementary Figure S2. Network Meta-Analysis of sensitivity analysis

Data in (a)and (b) are SMD (95% CrI) for the comparison of row-defining treatment versus column-defining treatment. SMD less than 0 favors upper-row treatment. Data in (c) are OR (95% CrI) for the comparison of row-defining treatment versus column-defining treatment. OR more than 1 favors upper-row treatment. Significant results are highlighted in bold; second line treatment are highlighted in blue; third line treatment are highlighted in yellow.

SNM: sacral neuromodulation; PTNS: peripheral tibial nerve stimulation

(a) micturition (upper triangle); UUI (lower triangle)

**SNM**	-0.27 (-1.14, 0.57)	-0.86 (-2.00, 0.24)	**-0.96 (-1.92, -0.02)**	**-1.00 (-1.95, -0.07)**	**-1.76 (-2.71, -0.84)**
**-0.30 (-0.57, -0.03)**	**BoNT-A**	-0.59 (-1.32, 0.12)	**-0.69 (-1.11, -0.27)**	**-0.73 (-1.13, -0.34)**	**-1.48 (-1.88, -1.10)**
**-1.34 (-2.26, -0.41)**	**-1.04 (-1.95, -0.12)**	**PTNS**	-0.10 (-0.72, 0.55)	-0.14 (-0.75, 0.49)	**-0.89 (-1.50, -0.27)**
**-1.49 (-1.96, -1.02)**	**-1.19 (-1.63, -0.74)**	-0.15 (-0.97, 0.67)	**Mirabegron**	-0.04 (-0.21, 0.12)	**-0.80 (-0.97, -0.63)**
**-1.48 (-1.93, -1.03)**	**-1.18 (-1.61, -0.75)**	-0.14 (-0.95, 0.67)	0.01 (-0.13, 0.15)	**Antimuscarinics**	**-0.76 (-0.87, -0.64)**
**-1.97 (-2.42, -1.51)**	**-1.67 (-2.09, -1.24)**	-0.63 (-1.44, 0.19)	**-0.47 (-0.61, -0.34)**	**-0.49 (-0.56, -0.41)**	**Placebo**

(b) UI (upper triangle); urgency (lower triangle)

**SNM**	0.28 (-0.90, 1.41)	0.21 (-1.90, 2.29)	-1.16 (-2.60, 0.23)	-1.01 (-2.45, 0.37)	**-1.68 (-3.10, -0.33)**
**-0.20 (-1.33, 0.89)**	**BoNT-A**	-0.07 (-1.85, 1.69)	**-1.43 (-2.30, -0.59)**	**-1.29 (-2.15, -0.45)**	**-1.96 (-2.78, -1.17)**
		**PTNS**	-1.37 (-2.97, 0.23)	-1.22 (-2.82, 0.38)	**-1.90 (-3.47, -0.32)**
**-1.48 (-2.42, -0.55)**	**-1.28 (-1.99, -0.54)**		**Mirabegron**	0.15 (-0.17, 0.46)	**-0.53 (-0.83, -0.23)**
**-1.31 (-2.18, -0.43)**	**-1.11 (-1.78, -0.40)**		0.17 (-0.16, 0.52)	**Antimuscarinics**	**-0.68 (-0.95, -0.39)**
**-2.21 (-3.11, -1.31)**	**-2.00 (-2.65, -1.34)**		**-0.73 (-1.03, -0.41)**	**-0.90 (-1.11, -0.70)**	**Placebo**

(c) 100% reductions from baseline in UIE/day (upper triangle); ≥50% reductions from baseline in UIE/day (lower triangle)

OnabotulinumtoxinA	3.55 (1.93, 6.78)	4.04 (2.36, 7.12)	4.01 (2.36, 7.04)	5.92 (3.53, 10.29)
1.41 (0.96, 2.08)	SNM	1.14 (0.57, 2.24)	1.13 (0.57, 2.19)	1.67 (0.84, 3.25)
2.17 (1.49, 3.17)	1.54 (0.94, 2.53)	Mirabegron	0.99 (0.85, 1.16)	1.47 (1.27, 1.69)
2.65 (1.81, 3.88)	1.88 (1.16, 3.06)	1.22 (0.97, 1.53)	Antimuscarinics	1.48 (1.29, 1.70)
3.57 (2.56, 5.00)	2.54 (1.59, 4.06)	1.65 (1.37, 1.98)	1.35 (1.08, 1.68)	**Placebo**

Supplementary Figure S3. Rank probabilities of sensitivity analysis in regard to (a) mean change in the frequency of micturition/day; (b) mean change in urgency urinary incontinence episodes (UUIE)/day; (c) mean change in urinary incontinence episodes (UIE)/day; (d) mean change in urgency/day; (e) ≥50% reductions from baseline in urinary incontinence episodes (UIE)/day; (f) 100% reductions from baseline in urinary incontinence episodes (UIE)/day.

These plots were made by GraphPad Prism 8.

SNM: sacral neuromodulation; PTNS: peripheral tibial nerve stimulation

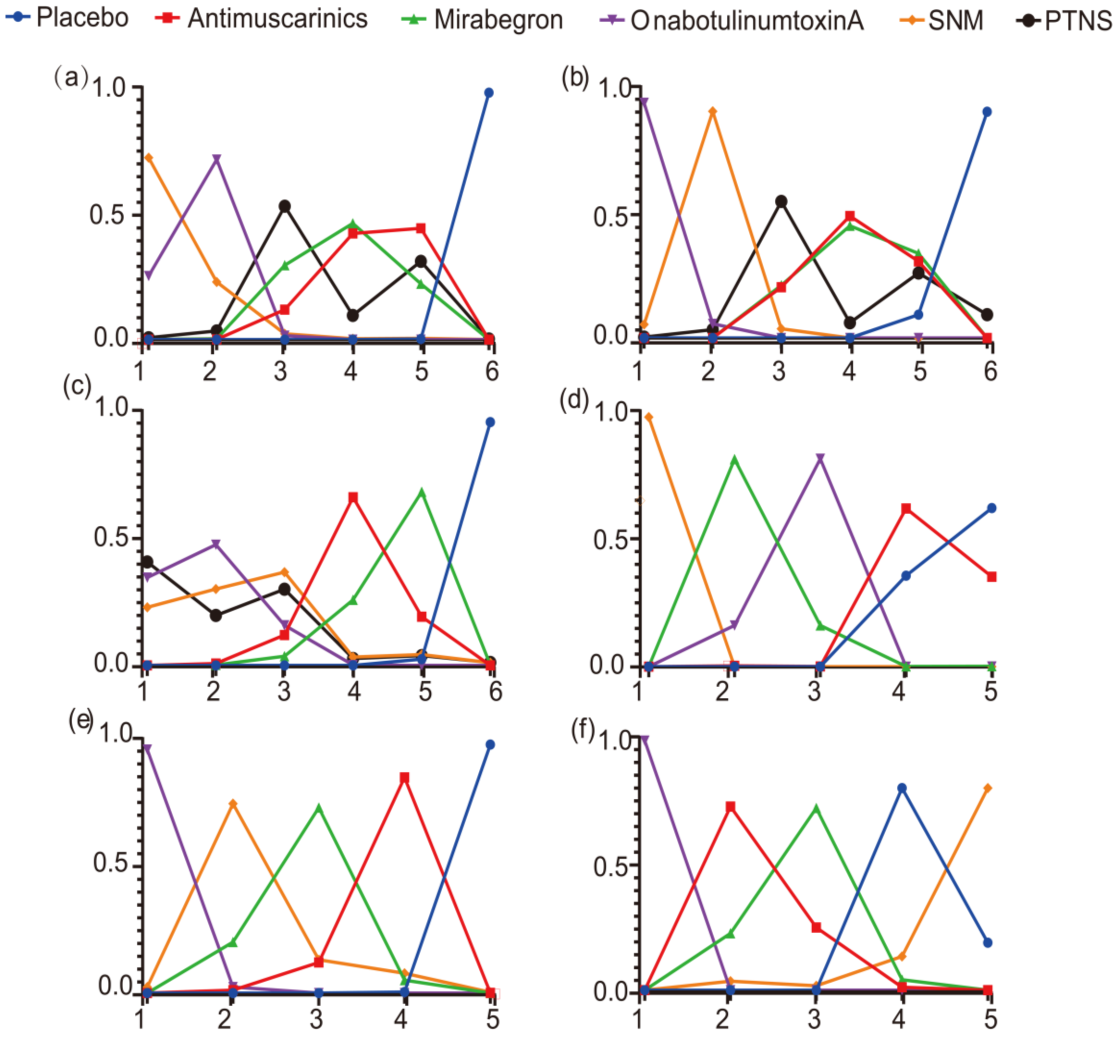

Supplement: Supplementary file 1 [file Data_Sheet_1.PDF]
